# Epiplakin expression dynamics during colon carcinogenesis: Correlation with proliferation

**DOI:** 10.17305/bb.2024.10981

**Published:** 2024-07-24

**Authors:** Damla Gül Fındık, Erhan Şahin, Özlem Türelik, Gürkan Güneri

**Affiliations:** 1 Department of Histology and Embryology, Faculty of Medicine, Bilecik Şeyh Edebali University, Bilecik, Türkiye; 2 Department of Pathology, Faculty of Medicine, Bilecik Şeyh Edebali University, Bilecik, Türkiye; 3 Department of General Surgery, Faculty of Medicine, Bilecik Şeyh Edebali University, Bilecik, Türkiye

**Keywords:** Colonic neoplasms, colonic polyps, neoplasms, plakins

## Abstract

Colorectal cancer poses a significant global health challenge, with a considerable proportion arising from colon adenomas. Understanding the molecules involved in the carcinogenesis process is crucial for improving colon cancer diagnosis and prognosis. While research on the role of epiplakin in cancer remains limited compared to other plakin group proteins, comprehending its expression patterns and correlations can offer valuable insights into colon carcinogenesis. In this study, we analyzed 60 tissue samples, including colon adenocarcinomas, tubular adenomas (low malignancy risk group), tubulovillous adenomas (high malignancy risk group), and adjacent normal colon tissues. Classification and grading were reevaluated by histological examination. Immunohistochemistry (IHC) was performed to assess epiplakin and Ki67 expression. Epiplakin optical density (OD) and the Ki67 proliferation index were calculated using ImageJ. Statistical analyses were conducted to evaluate correlations and significance. Epiplakin expression was significantly decreased in colon adenocarcinomas [OD median 4.04 (95% CI, 3.98–4.24)] and tubulovillous adenomas [4.32 (95% CI, 4.08–4.32)] compared to normal colon tissues [4.61 (95% CI, 4.50–4.67)] and tubular adenomas [4.87 (95% CI, 4.67–4.88)] (*P* < 0.05). Moreover, adenoma groups exhibited higher proliferation indices (*P* < 0.05), and a positive correlation was found between epiplakin expression and the Ki67 proliferation index (*r* ═ 0.317, *P* < 0.05). Our study highlights the potential significance of epiplakin in colorectal cancer. Decreased epiplakin expression is associated with colon malignancy progression, suggesting its role as a potential marker.

## Introduction

Colon cancer stands as a prominent public health concern on a global scale. When examining the landscape of cancer statistics worldwide, colon cancer emerges as the third most commonly diagnosed cancer and the second leading cause of cancer-related mortality [1]. From an epidemiological perspective, it is important to note that recently, a new subset of cancers of unknown primary site (CUP) with a colon-cancer profile (CUP-CCP) has emerged. CUP-CCP is characterized by adenocarcinoma with features suggestive of colorectal origin, primary intra-abdominal metastases, and specific immunohistochemical staining [cytokeratin (CK) 20+, CDX2+, CK7−]. This subset, treated as colon cancer, contributes to the increased incidence of colon cancer. CK20 and CDX2 are highly specific markers for colorectal carcinomas, with CK20 expressed in 70%–100% of cases and CDX2 in 97% [2]. In early-stage colon cancer, surgical intervention alone can be curative. For stage I colon cancer, the standard treatment involves surgery without additional therapies, achieving a 5-year survival rate of 95%. At this stage, adjuvant chemotherapy is generally not recommended. For stage II colon cancer, the 5-year survival rate with surgery alone ranges from 82% to 88% [3]. Therefore, early diagnosis of colon cancer is crucial for its manageability [4]. At this point, it is important to highlight that immune cell PD-L1 expression is significantly higher in mismatch repair (MMR)-deficient (MSI-H) colon cancer compared to MMR-proficient (MSI-L) tumors, with no differences observed among different MSI-H molecular subtypes. Recommended screening for defective DNA MMR includes immunohistochemistry (IHC) and/or microsatellite instability (MSI) testing. However, challenges remain in translating the biological and technical heterogeneity of MSI testing into useful clinical information. Literature reports suggest that IHC testing of the MMR mechanism may yield different results for a given germline mutation, potentially due to somatic mutations [5]. A notable proportion of colon adenocarcinomas originate from colon adenomas, which are characterized by dysplastic epithelial growth [6]. Tubular adenomas carry a malignancy risk of 5%, whereas tubulovillous adenomas in the intermediate-risk group have a higher malignancy risk of 22% [7]. Understanding the molecules that may play a role in carcinogenesis during the development of carcinomas from polyps is essential for improving the diagnosis and prognosis of colon cancer.

The process of carcinogenesis is closely associated with cell proliferation, invasion, and adhesion, all of which involve the dynamic roles of cell cytoskeleton molecules [8]. Tumor cells establish a microenvironment that plays a crucial role in influencing cancer progression. This microenvironment is shaped by complex biochemical and biophysical interactions between tumor cells, stromal cells, and the extracellular matrix. Cells attach to the tumor microenvironment through cell cytoskeletons, which transform external mechanical signals into cellular responses, such as invasion and migration [9]. Actin cytoskeleton organization is critical for the differentiation of epithelial cells into mobile mesenchymal cells, a process known as epithelial–mesenchymal transition, which is important in cancer progression [10]. Microtubules, another component of the cell cytoskeleton, are functional in migration processes other than the amoeboid invasion of cancer cells [11].

Plakins are molecules that facilitate connections between the microfilaments, intermediate filaments, and microtubules within the cell’s cytoskeleton, particularly at cell junctions. Considering the significant role of the cell cytoskeleton in carcinogenesis, there is a hypothesis that binding proteins like plakins may also contribute to this process. Studies have indicated that plakins, including plectin, periplakin, and desmoplakin, could serve as valuable biomarkers in cancer contexts [8]. For instance, high levels of plectin have been associated with prostate cancer, regulating cancer growth and metastasis, while the inhibition of periplakin in pharyngeal squamous cancer cells reduces cellular movement and adhesion [12, 13]. Additionally, low desmoplakin expression is linked to an increased risk of distant metastasis in oropharyngeal squamous cell cancer [14].

Epiplakin, another plakin protein, is expressed in various tissues, such as the esophagus, colon mucous epithelial cells, stomach, epidermis, parotid, and sweat glandular cells [15]. It possesses unique characteristics, notably lacking the N-terminal region found in other plakin proteins [8]. However, the relationship between epiplakin and cancer remains largely unexplored. Loss of epiplakin expression has been associated with advanced pancreatic cancer, while in cervical cancer, it has been found to increase cell proliferation through the p38 signaling pathway, correlating with tumor size [16, 17]. Despite this, the literature lacks studies investigating epiplakin expression in colon cancer and adenomas. An analysis of GEPIA2 data indicates an increase in epiplakin gene expression in colon adenocarcinoma compared to normal tissue. In contrast, UALCAN data suggest a decrease in epiplakin protein expression in colon adenocarcinoma compared to normal tissue [18, 19]. These data imply a potential role for epiplakin in colon carcinogenesis. Thus, this study aims to explore changes in epiplakin expression during the carcinogenesis process from colon polyps to carcinomas, a crucial aspect of the cell cytoskeleton. Additionally, our research seeks to evaluate the relationship between epiplakin and the proliferation step of colon carcinogenesis by assessing the correlation between epiplakin and Ki-67, a proliferation marker in colon polyps and carcinomas.

## Materials and methods

### Tissue sample collection, inclusion, and exclusion criteria

The samples analyzed were sourced from the pathology department archives of Bilecik Training and Research Hospital, covering the period from 2015 to 2022. They comprised paraffin-embedded blocks and hematoxylin-eosin stained slides. The samples included 15 low-grade tubular adenomas, 15 low-grade tubulovillous adenomas, 15 moderately differentiated colon adenocarcinomas, along with 15 adjacent normal colon tissues.

Patients included in the study had not undergone prior radiotherapy or chemotherapy and had a primary focus on the colon. To ensure that the analysis results were not influenced by external factors, individuals with skin or lung diseases were excluded from the study. This exclusion was particularly important given the role of plakin-associated proteins in skin keratinocyte adhesion and their association with bronchiolitis obliterans [20, 21]. For the cancer group, we selected patients with moderately differentiated colon adenocarcinomas. For the adenoma groups, we included cases without a history of cancer and those with isolated low-grade tubular or tubulovillous adenomas. High-grade adenomas showing dysplasia were excluded to avoid statistical skewing, as they are less commonly observed.

### Histochemistry

Histological classification and grading of tissue samples stained with hematoxylin and eosin were reevaluated by a histologist and pathologist. Colon cancer presents with three main histopathological types: adenocarcinoma, mucinous adenocarcinoma, and signet ring cell carcinoma. Among these, adenocarcinoma is the most common and generally has a milder clinical course compared to the others [22]. According to the current cancer protocol of the College of American Pathologists (CAP), colon cancer is categorized into four grades. Grade 1 represents the most well-differentiated group, characterized by well-structured tubules, minimal nuclear polymorphism, and a high percentage of gland formation (>95%). Grade 3, on the other hand, is the least differentiated group with few glandular structures, high nuclear polymorphism, and a low percentage of gland formation (<50%). Grade 2 falls in between, representing a moderately differentiated group with 50%–95% gland formation [23]. In our study, Grade 2 – moderately differentiated colon adenocarcinoma was compared with benign lesions, adenomas (tubular and tubulovillous adenomas), in terms of evaluating carcinogenesis. Colon adenomas are categorized into three histological types: tubular adenomas (<25% villous component), tubulovillous adenomas (25%–75% villous component), and villous adenomas (>75% villous component) [24]. In our study, low malignant (5% malignancy risk) tubular and moderately malignant (22% malignancy risk) tubulovillous adenomas were investigated to reveal potential differences compared to malignant adenocarcinoma [7, 25, 26]. All selected tubular or tubulovillous adenomas were low grade.

**Table 1:** Clinicopathologic features of the groups

**Gender**	**Male, *n* (%)**	**Female, *n* (%)**	**Total, *n* (%)**		
N	9 (60)	6 (40)	15 (100)		
T	10 (66.7)	5 (33.3)	15 (100)		
TV	5 (33.3)	10 (66.7)	15 (100)		
CA	7 (46.7)	8 (53.3)	15 (100)		
Total	31 (51.7)	29 (48.3)	60 (100)		
**Age**	**Mean**	**Std. Deviation**	*P*		
N	64.13	8.83	0.796		
T	61.13	12.18			
TV	60.47	11.83			
CA	61.33	9.81			
Total	61.77	10.57			
**Localization**	**Cecum (%)**	**Ascending colon (%)**	**Transvers colon (%)**	**Descending colon (%)**	**Sigmoid colon (%)**
N	26.7	13.3	26.7	33.3	0
T	6.7	20	13.3	13.3	46.7
TV	13.3	13.3	13.3	46.7	13.3
CA	20	13.3	20	20	26.7
Total	16.7	15	18.3	28.3	21.7

Oneway ANOVA test; *P* > 0.05. Std: Standard; N: Adjacent normal tissue; T: Tubular adenoma; TV: Tubulovillous adenoma; CA: Cancer group.

### Immunohistochemistry

In immunohistochemical staining, 4 µm sections from paraffinized tissue blocks underwent deparaffinization, followed by hydration through a decreasing alcohol series. Antigen retrieval was achieved by incubating the sections in pH 6 citrate buffer. To block endogenous peroxidase activity in the tissue, 0.3% hydrogen peroxide was applied. Subsequently, the sections were incubated overnight with a 1:200 diluted epiplakin primary antibody (EPPK1, Thermo, PA5-64412) or a 1:100 diluted Ki-67 primary antibody (Thermo, RM-9106-S0). Afterward, secondary antibody (Epredia, UltraVision Detection System) incubation was performed. Immunoreactivity was detected using streptavidin-HRP (Epredia, UltraVision Detection System) and chromogen (Epredia, DAB chromogen kit). Human skin samples served as positive controls for epiplakin, while tonsil tissue samples were used as positive controls for Ki-67. Images were captured under a light microscope (Olympus CX23) with an attached camera (Olympus EP50, 1920 (W) × 1080 (H) pixels).

### Quantitative analysis of images

Immunohistochemical images were processed using ImageJ software (Image J v1.53e), and areas of chromogen staining were segmented using the color deconvolution function. Quantitative analysis was conducted for the average immunoreactivity intensity. For each sample, ten fields at ×400 magnification were evaluated [27]. Following deconvolution, chromogen-stained areas were selected using the threshold function, and the number of pixels within these areas was calculated. For EPPK1 intensity, integrated optical density (IOD) was calculated as a logarithmic value. For Ki-67, the fractional (%) stained area relative to the total image area was used to calculate the Ki-67 index [28].

### Ethical statement

Ethical approval for this study was obtained from the Ethics Committee of Bilecik University (approval number: 2023/1-7), ensuring adherence to the ethical guidelines specified in the Declaration of Helsinki. Formal written informed consent was not required due to the retrospective design of the study, as waived by the appropriate IRB.

### Statistical analysis

Statistics program (IBM SPSS Statistics, version 26.0) was used for statistical analysis. The normal distribution of groups was assessed using the Kolmogorov–Smirnov test. One-way ANOVA was used for age statistics. To compare epiplakin expression between groups, the Kruskall–Wallis test for non-parametric data analysis was employed. A *P* value less than 0.05 was considered statistically significant in all analyses. Significance values were adjusted using the Bonferroni correction for multiple tests. The impact of epiplakin on Ki-67 expression was evaluated through correlation analysis. Spearman’s non-parametric test was utilized to assess the correlation between epiplakin and Ki-67.

## Results

### Clinicopathologic results

The study included a total sample size in which 51.7% were male, indicating a slightly higher prevalence in males compared to females. The participants had a mean age of 61.77 ± 10.57 years, reflecting the typical age range for the onset of colon cancer. Statistical analysis showed no significant differences in age distribution among the different groups. When examining the localization within the colon, it was found that the left colon, which includes the descending colon and sigmoid colon, was more frequently affected (Table 1).

### Histopathological results

**Figure 1.**
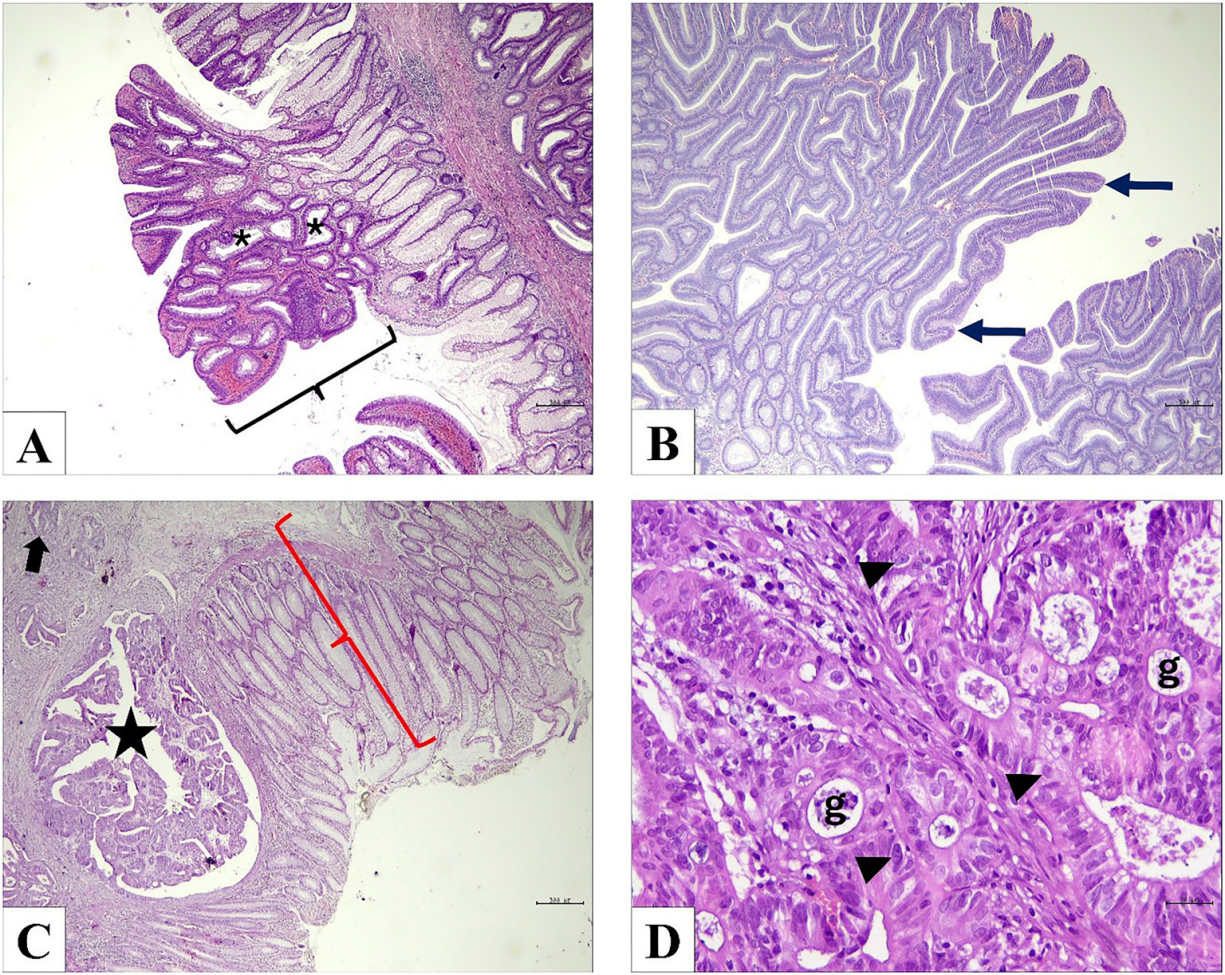
**Histological examination of the groups with hematoxylin and eosin staining.** (A) Tubular adenoma group; polypoid growth pattern (black parentheses), gland structures consisting of hyperchromatic nuclei and pseudostratified epithelial cells (*) (×40); (B) Tubulovillous adenoma group; villous growth pattern containing pseudostratified epithelial cells with hyperchromatic nuclei (thin arrow) (×40); (C) Adenocarcinoma and adjacent normal tissue; adjacent normal intestinal mucosa (red parentheses), cancer tissue with irregular gland structures containing hyperchromatic nuclei (star), infiltration (thick arrow) (×40); (D) Adenocarcinoma group; pleomorphic cells with large nuclei, distinct hyperchromatic nucleoli (arrowhead), and irregular gland structures showing occasional cribriformity (g) (×400).

Upon histological examination, distinct glandular structures were identified within each group of tissue samples. In the tubular adenoma group, these gland structures exhibited a characteristic polypoid growth pattern, composed of pseudostratified epithelial cells with hyperchromatic nuclei (Figure 1A). In contrast, the tubulovillous adenoma group displayed a more complex architecture, featuring both tubular and villous growth patterns. Within this group, the presence of villous structures alongside pseudostratified epithelial cells added to the histopathological diversity observed (Figure 1B). Moving on to the adenocarcinoma group, irregular and infiltrative gland structures were noted, comprised of hyperchromatic nuclear cells indicative of dysplastic changes. Notably, occasional areas of adjacent normal intestinal mucosa were identified (Figure 1C). The adenocarcinoma was moderately differentiated and exhibited features consistent with a Grade 2 classification. Pleomorphic cells with large nuclei and distinct hyperchromatic nucleoli were prominent, along with irregular gland structures displaying occasional cribriformity (Figure 1D).

### Epiplakin expression results

**Figure 2.**
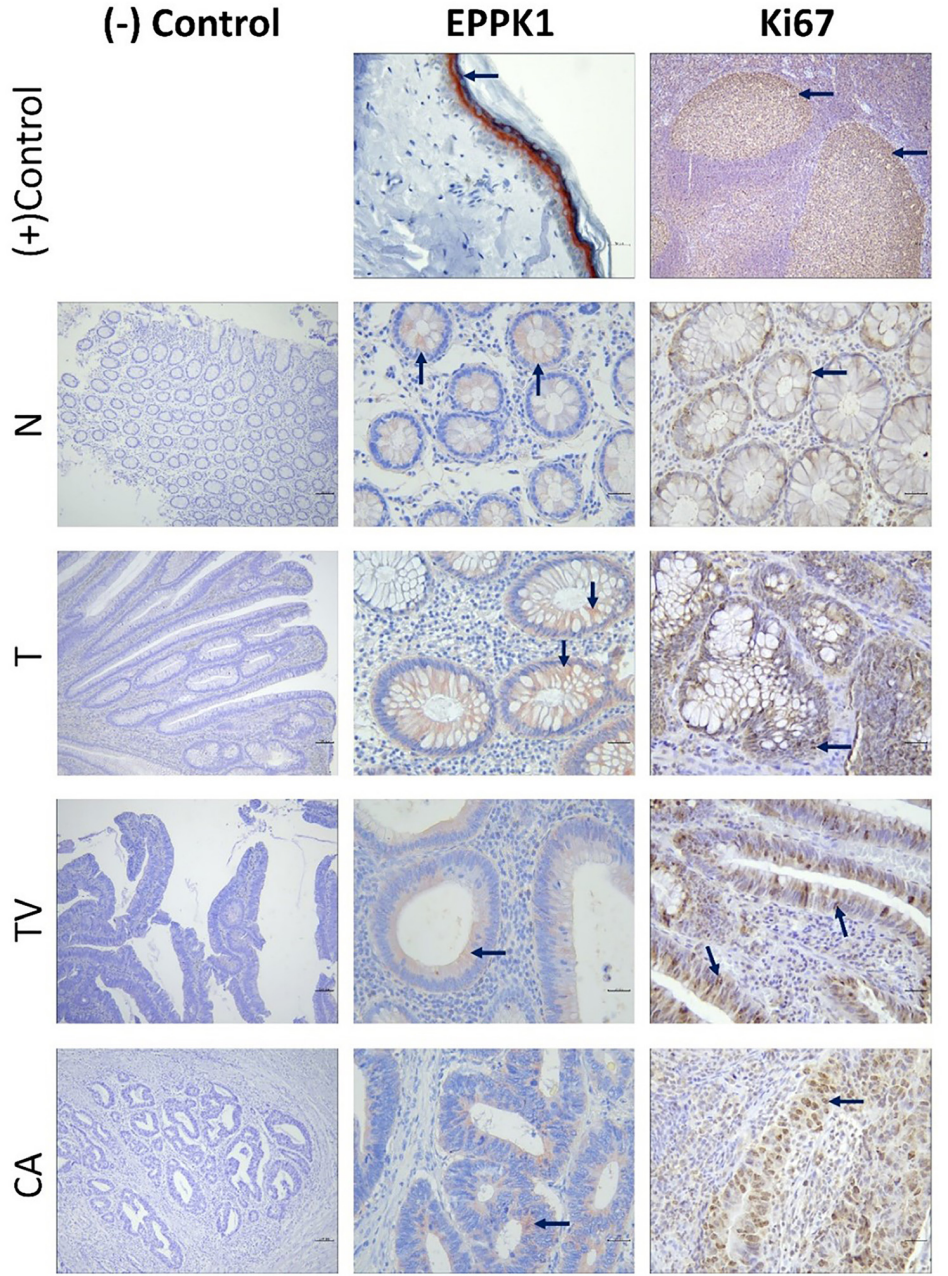
**Epiplakin (EPPK1) and Ki67 immunohistochemical staining of the groups with positive controls.** The first panels of immunohistochemical stains present negative controls (×100). Immunoreactivity is represented with a black arrow. (EPPK1) Positive control for EPPK1 is human skin tissue, demonstrating intense cytoplasmic expression in the stratum granulosum layer with mild expression in the stratum spinosum. EPPK1 shows cytoplasmic expression in the glandular epithelium of the experimental groups (Eppkx400). (Ki67) Positive control for Ki67 is human tonsil tissue, displaying predominant nuclear expression in germinal centers (Ki67x100). Ki67 shows predominantly nuclear expression, occasionally accompanied by mild cytoplasmic expression in the experimental groups (Ki67x400). N: Adjacent normal tissue; T: Tubular adenoma; TV: Tubulovillous adenoma; CA: Cancer group.

**Figure 3.**
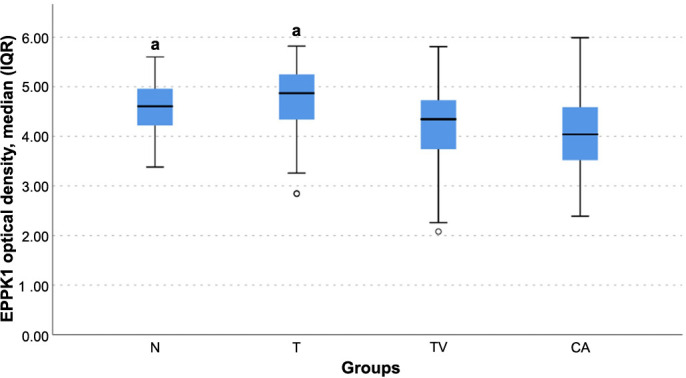
**Boxplot graph of EPPK1 immunoreactivity assessment via optical density of the groups.** Kruskal–Wallis test and post-hoc Bonferroni, *P* < 0.05. a: Significant difference compared with TV and CA groups. IQR: Interquartile range; N: Adjacent normal tissue; T: Tubular adenoma; TV: Tubulovillous adenoma; CA: Cancer group.

Immunohistochemical staining of epiplakin revealed distinct patterns of expression within the different tissue samples. In the positive control (skin), intense epiplakin expression was observed in the stratum granulosum layer, with milder cytoplasmic expression in the stratum spinosum. In the colon tissue samples, cytoplasmic expression of epiplakin was primarily observed in the glandular epithelium. However, a notable decrease in cytoplasmic epiplakin expression was evident in both tubulovillous adenomas and colon adenocarcinomas compared to other groups (Figure 2). Quantitative analysis of epiplakin expression revealed statistically significant differences in optical density (OD) between the groups (Figure 3). Specifically, tubulovillous adenomas [4.32 (95% CI, 4.08–4.32)] and adenocarcinomas [4.04 (95% CI, 3.98–4.24)] exhibited lower epiplakin densities compared to normal [4.61 (95% CI, 4.50–4.67)] and tubular adenomas [4.87 (95% CI, 4.67–4.88)] (*P* < 0.05) (Table 1). The decrease in epiplakin density was notable in tubulovillous adenomas, which bear a higher malignancy risk compared to tubular adenomas (Figure 3). These findings suggest a potential association between epiplakin expression and malignancy.

### Ki67 expression results

Immunohistochemical staining for Ki67 revealed predominantly nuclear expression, occasionally accompanied by mild cytoplasmic staining. Intense Ki67 expression was observed in the germinal centers of the tonsil tissue, serving as the positive control (Figure 2). Evaluation of proliferation indices demonstrated statistically higher Ki67 indices in adenoma groups [tubular adenoma 9.77 (95% CI, 9.61–11.80), tubulovillous adenoma 9.41 (95% CI, 8.91–10.76)] compared to both normal [6.96 (95% CI, 7.16–8.77)] and cancer groups [5.46 (95% CI, 6.55–8.99)] (*P* < 0.05). Notably, the Ki67 index of the Grade 2 moderately differentiated cancer group did not significantly differ from adjacent normal tissue (Table 2).

**Table 2:** EPPK1 OD and proliferation index (%Ki67) parameters belonging to the groups with pairwise comparisons

**OD of EPPK1**	**Median**	**%95 CI**		
		**Lower bound**	**Upper bound**	
N	4.61	4.50	4.67	
T	4.87	4.67	4.88	
TV	4.32	4.08	4.32	
CA	4.04	3.98	4.24	
**Pairwise comparisons**	**Test statistic**	**Std. error**	**Std. test statistic**	***P* ^a^**
CA-TV	26.90	20.02	1.3	1
CA-N	109.22	20.02	5.46	0.00*
CA-T	158.78	20.02	7.93	0.00*
TV-N	82.32	20.02	4.11	0.00*
TV-T	131.88	20.02	6.59	0.00*
N-T	−49.56	20.02	−2.48	0.08
**%Ki67**	**Median**	**%95 CI**		
		**Lower bound**	**Upper bound**	
N	6.96	7.16	8.77	
T	9.77	9.61	11.80	
TV	9.41	8.91	10.76	
CA	5.46	6.55	8.99	
**Pairwise comparisons**	**Test statistic**	**Std. error**	**Std. test statistic**	***P* ^a^**
CA-N	34.81	20.02	1.74	0.49
CA-TV	90.22	20.02	4.51	0.00*
CA-T	100.74	20.02	5.03	0.00*
N-TV	−55.41	20.02	−2.77	0.03*
N-T	−65.93	20.02	−3.29	0.006*
TV-T	10.52	20.02	0.53	1

Kruskal–Wallis test. *P < 0.05, a: Adjusted significance value by the Bonferroni correction for multiple tests. CI: Confidence Interval; Std: Standard; N: Adjacent normal tissue; T: Tubular adenoma; TV: Tubulovillous adenoma; CA: Cancer group; OD: Optical density.

### Relationship between epiplakin and Ki67

**Figure 4.**
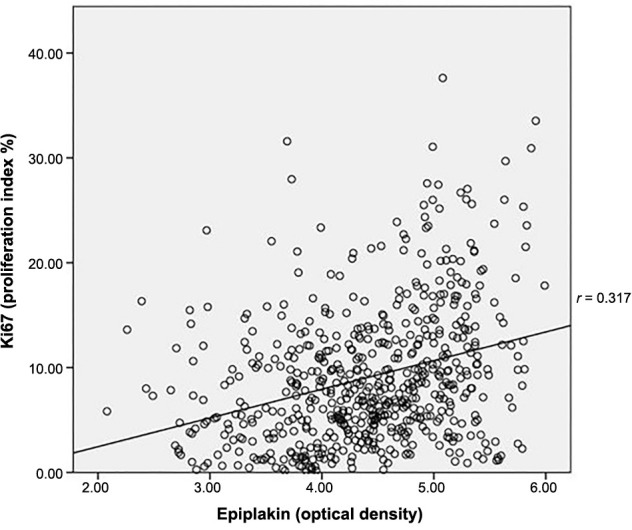
**Scatter/dot graph of optical density of EPPK1 and proliferation index (Ki67).** Spearman test, *r*: Correlation coefficient, *P* < 0.05. OD: Optical density.

A positive correlation was identified between epiplakin OD and Ki67 proliferation index (*P* < 0.05), characterized by a correlation coefficient of 0.317. This positive correlation suggests a potential link between epiplakin expression and cell proliferation within the context of colorectal carcinogenesis (Figure 4).

## Discussion

Plakin molecules have emerged as potential biomarkers in cancer research. Among these, epiplakin stands out due to its unique structure compared to other plakin group proteins. However, there is a scarcity of studies investigating its involvement in cancer [8]. Notably, our study fills this gap by examining epiplakin expression in colon cancer and adenomas, shedding light on its potential role in carcinogenesis. Different data underscore the complexity of epiplakin regulation in cancer contexts [18, 19].

Fujiwara et al. [29] demonstrated epiplakin expression in colon mucous epithelial cells using immunofluorescence, a finding consistent with our study. Additionally, Yoshida et al. [16] observed a decrease in epiplakin expression associated with the loss of epithelial ductal characteristics in late-stage pancreatic cancer. Among other plakin proteins, periplakin expression has been reported to decrease in urothelial carcinoma of the bladder [30]. Additionally, in colon cancer cases, periplakin has been found to decrease in relation to tumor size [31]. The inclusion of adenoma cases in our study allowed for a comprehensive assessment of carcinogenesis stages, revealing a consistent decrease in epiplakin expression across both colon adenocarcinoma and tubulovillous adenomas (high malignancy risk group) (*P* < 0.05). This underscores the potential significance of epiplakin for malignancy progression in colon cancer.

The inhibition of another plakin protein, desmoplakin, has been reported to activate the WNT/β-catenin pathway and epithelial–mesenchymal transition in gastric cancer cells [32]. In addition, desmoplakin has been shown to act as a tumor suppressor by activating the WNT/β-catenin pathway in non-small cell lung cancer studies [33]. Decreased desmoplakin expression is also associated with breast cancer metastasis and invasion [34]. The decreased expression of epiplakin associated with malignancy potential, as observed in our study, may be correlated with the activation of epithelial–mesenchymal transition, similar to the findings reported for desmoplakin in gastric, lung, and breast cancers. Furthermore, plectin-deficient human hepatic cells display elevated cell motility, accompanied by an increase in focal adhesion kinase activity, which closely resembles the characteristics observed in invasive hepatocellular carcinoma [35]. These findings suggest a potential role for plakins in modulating cell motility and highlight their significance in carcinoma progression.

Shyskin observed an increase in Ki67 expression from adenomas to adenocarcinomas, indicating high proliferation in the early stages of colorectal carcinogenesis. This suggests that there is heightened proliferation in the premalignant early stages of colorectal carcinogenesis [36]. Consistent with these findings, our study observed an elevated Ki67 index in low-grade tubular and tubulovillous adenomas compared to both normal and cancerous tissues (*P* < 0.05). Additionally, Heidari et al. [37] found higher Ki67 expression in the cancer group compared to normal tissue in a sample predominantly consisting of well-differentiated and mucinous adenocarcinomas. However, our study did not detect a significant difference in Ki67 expression between tumor-adjacent normal tissue and cancer tissue in non-mucinous adenocarcinomas. This discrepancy may be due to differences between non-mucinous and mucinous adenocarcinomas. Tong et al. [38] reported a positive correlation of 0.456 between tumor differentiation and Ki67 expression in colorectal cancer. However, our study focused exclusively on moderately differentiated cancers and lacked high-grade lesions, which may have contributed to the observed lower Ki67 expression levels.

While most studies focus on plektin within the plakin group, our study underscores the importance of investigating epiplakin’s role in cancer [12]. In a study by Perez et al. [39], a monoclonal plektin antibody inhibited growth in ovarian cancer. Additionally, a study on cervical cancer related to epiplakin found that it increased cell proliferation via the p38 signaling pathway and showed a correlation with tumor size [17]. Consistent with these findings, our study identified a correlation between epiplakin expression and Ki67, a proliferation marker (*r* ═ 0.317, *P* < 0.05). Melling et al. [40] demonstrated that increased Ki67 expression in colorectal cancers is associated with low tumor stage and is a good prognostic marker. Accordingly, epiplakin, which has been shown to correlate with Ki67, could serve as a promising prognostic marker in colorectal cancer. Further studies are warranted to investigate this possibility.

Karabulut et al. [41] found that colon cancer occurred in 70% of males and was located in the left colon in 80% of cases. There are different rates of mutations in key oncogenes and tumor suppressors between right- and left-sided colon cancer [42]. For instance, Bylsma et al. [43] found a significant association between the *BRAF* V600E mutation and right-sided colon cancer, along with a higher prevalence of *KRAS* mutations in these cases. Conversely, mutations in *APC* and *TP53* are more common in left-sided colon cancer. Amplifications of receptor tyrosine kinases (e.g., *ERBB2*) and higher expressions of epidermal growth factor receptor *(EGFR*) and its ligands, epiregulin (*EREG*) and amphiregulin (*AREG*), are also more prevalent in left-sided cancers [42]. Tran et al. [44] found that right-sided tumors are associated with worse overall survival compared to left-sided ones, suggesting that laterality may influence genetic profiles and act as an independent prognostic factor. Colon cancer peaks after the age of 60, but there is no consensus on whether age affects survival outcomes. Prognosis in older patients may be affected by differences in stage at presentation, tumor site, preexisting comorbidities, and treatment type [45, 46]. Our study findings align with the existing literature. However, since the rectum was not included in our analysis, the percentages observed in our study were lower.

## Conclusion

In summary, our study emphasizes the potential significance of epiplakin in colon cancer. Despite the limited research on epiplakin in cancer, our findings suggest a connection between decreased epiplakin expression and the progression of colon malignancy. We observed a positive correlation between epiplakin expression and the Ki67 index, indicating increased proliferation. However, its potential prognostic value in colorectal cancer needs further investigation, warranting larger, multicenter studies. The current study underscores the importance of evaluating epiplakin alongside other plakin proteins in cancer research.

## Data Availability

The data that support the findings of this study are available from the corresponding author upon reasonable request.
